# Does a medical management program for CKD patients postpone renal replacement therapy and mortality?: A 5-year-cohort study

**DOI:** 10.1186/1471-2369-13-138

**Published:** 2012-10-22

**Authors:** Mitra Mahdavi-Mazdeh, Zinat Nadia Hatmi, Sara Shahpari-Niri

**Affiliations:** 1 Iranian Tissue Bank Research & Preparation Center, Tehran University of Medical Sciences, Tehran, Iran; 2 Research Center of Nephrology, Tehran University of Medical Sciences, Tehran, Iran; 3 Tehran University of Medical Sciences, Tehran, Iran

## Abstract

**Background:**

Many countries have started screening and prevention programs for chronic kidney disease (CKD). However, one of the main concerns of health authorities is whether management strategies for diagnosed CKD patients can decrease mortality or morbidity. This study aimed to investigate the effect of two competing clinical strategies of treatments under nephrologists’ supervision compared with no treatment on the frequency of the need to start renal replacement therapy (RRT) and mortality in CKD patients.

**Methods:**

Our cohort comprised consecutive newly diagnosed patients with CKD in an outpatient clinic in Tehran between October 2002 and October 2011. CKD Patient enrollment occurred if two criteria of high plasma creatinine level and chronicity of renal disease by at least 3 months of clinical history or small sized kidneys in ultrasound findings were met. Demographic data and time of RRT or mortality in patients who had been followed up regularly were compared with those in the control group. The control group included those patients who did not attend a nephrology clinic to receive CKD management package for at least 1 year during the study period.

**Results:**

The cohort included 76 patients in the control group and 389 patients in the supervised group. The mean age of the patients was 61.33±14.9 years (16–95 years). The ratio of males/females was 1.47 (277/188). The mean follow-up in the control and supervised groups was 33.29±20.50 (7–111) and 36.03±25.24 (6–124) months , respectively, and the total patient years of follow-up was 1382.3. A substantial number of patients survived without RRT until the first year of follow up (96%) in both groups, but afterward, those in the control group had more deaths or need to start RRT in comparison with those who received medical advice (20 vs. 67 months; p= 0.029). This cohort also showed a higher survival and a longer time to show a GFR of less than 15 cc/min (84 vs 34 months, p<0.0001) in patients who had been under physician supervision compared with the control group.

**Conclusions:**

Active follow-up of CKD patients appears to significantly decrease the risk of death or progression to end-stage renal disease and the requirement to start renal replacement therapy.

## Background

The incidence and prevalence of end-stage renal disease (ESRD), which is not restricted to the developed world, is escalating. It is estimated that 80% of death due to chronic diseases is in developing countries, and 70% of the ESRD population will be found in this part of the world by 2030 [1-3]. The cost of renal replacement therapy (RRT) is huge, even in wealthy countries, such as the United States, where the ESRD population consumes 6-7% of the Medicare budget [3,4]. Similarly, in Taiwan, ESRD patients, which comprise 0.23% of the population, are allocated 7.2% of health-care resources. [5]It has also been shown that each month of dialysis in Iran costs at least 1000 USD [6].

International efforts through numerous campaigns such as World Kidney Day have made the medical community aware of the common frequency of chronic kidney disease (CKD), its strong association with diabetes, and higher attributed mortality [7]. Because screening without follow-up is of little use, many countries have started screening and prevention programs to tackle the increasing burden, which is mainly due to higher premature mortality and disability because of progression to ESRD [5,8-10]. However, one of the main concerns of health authorities is the quantitative effect of medical care of detected CKD patients after diagnosis in postponing or prevention of these outcomes.

The current cohort study aimed to determine if provision of a management program, including health education, diet modification, and pharmacological treatment through nephrology clinics for CKD patients, is able to reduce mortality and the need for RRT.

## Patients & methods

Our study sample comprised all newly diagnosed patients with CKD consecutively admitted to the authors' out-patient nephrology office or clinic in Tehran between October 2002 and October 2011. These patients were admitted of their own will for different reasons, or admission was based on recommendation of another physician. There were two renal outpatient clinics that offered diagnostic and follow-up services to patients with renal disease by just one responsible nephrologist (author,M.Mahdavi-Mazdeh). CKD patient enrollment took place if there were at least two separate calculated estimated glomerular filtration rate (eGFR) between 15 and 60 mL/min/1.73 m2 by serum creatinine and chronicity, determined by more than 3 months of clinical history or small sized kidney in ultrasound. Patients who did not complete their follow-up for at least 6 months were excluded from analysis.

To compare the effect of two clinical strategies of medical supervision and no treatment on the need to start RRT and/or mortality rate, two groups were defined. The first group included those patients who had been followed up regularly (389 patients) and the second group was the control group who did not attend the nephrology clinic for at least 1 year (76 patients). Therefore, we did not use an unethical approach to prevent some patients from receiving treatment. Finally, those patients followed up regularly (at least once every 6 months) were compared with those who failed to be followed up as frequently. If a patient did not attend the clinic for more than 1 year, he/she was included in the control group. Otherwise, those patients who used the prescriptions but attended the clinic with less frequent intervals, were included in the treated group.

Visits were scheduled according to each patient’s clinical setting, but were carried out at least every 6 months. During each visit, patients were monitored for body weight, blood pressure, serum creatinine levels, fasting plasma glucose levels, lipid profile, calcium, phosphorus, hemoglobin (Hb), and other parameters. All laboratory variables were collected in all patients for clinical purposes.

A total number of 465 patients were enrolled. The end points of the study were defined as time to RRT, time to GFR less than 15cc/min/m^2^, and time to death or reaching the deadline of data collection for the first step of analysis.

The treatment program consisted of drugs for blood pressure, bone mineral metabolism indices, and hemoglobin control. Vaccination for Hepatitis B virus according to each patient situation was scheduled. The two main parts of each visit apart from routine prescription were: (1) nutritional consultation regarding salt and water restriction, phosphate content of different foods, and protein consumption: and (2) checking for treatment compliance of patients with their accompanying persons (usually first degree relatives) and some advice to increase the compliance of patients. The goals of treatment protocols were based on Disease Outcomes Quality Initiative (KDOQI) guidelines. The routine protocol for anemia management in Iran includes recombinant α-erythropoietin and iron sucrose for intravascular iron and other different forms for the oral route. The α-erythropoietin was not covered by insurance agencies in the first 3 years of study, but after this time, those who required the drug could purchase it at a reasonable price. The phosphate binder was calcium carbonate. The main antihypertensives for all patients, except those with complications, were angiotensin-converting-enzyme inhibitors/angiotensin receptor blockers. All patients were advised to be vaccinated against Hepatitis B virus, and antibody titer was checked in following visits. Arteriovenous fistula was recommended for patients who were anticipated to need dialysis in the following 6–12 months.

### Statistical analysis

Descriptive data at the first assessment and follow-up are presented as mean± SD. Continuous and categorical variables were compared between the two groups by using the independent samples t-test and Pearson chi-squared test. Clinical end points were compared between groups by conducting survival analysis. Multivariate adjusted relations were evaluated by using the Cox proportional hazard model. All statistical analyses were performed by SPSS statistical package, version 16.0 (SPSS, Inc., Chicago,IL). A p less than 0.05 from 2-sided tests was considered significant.

## Results

**Figure 1:**
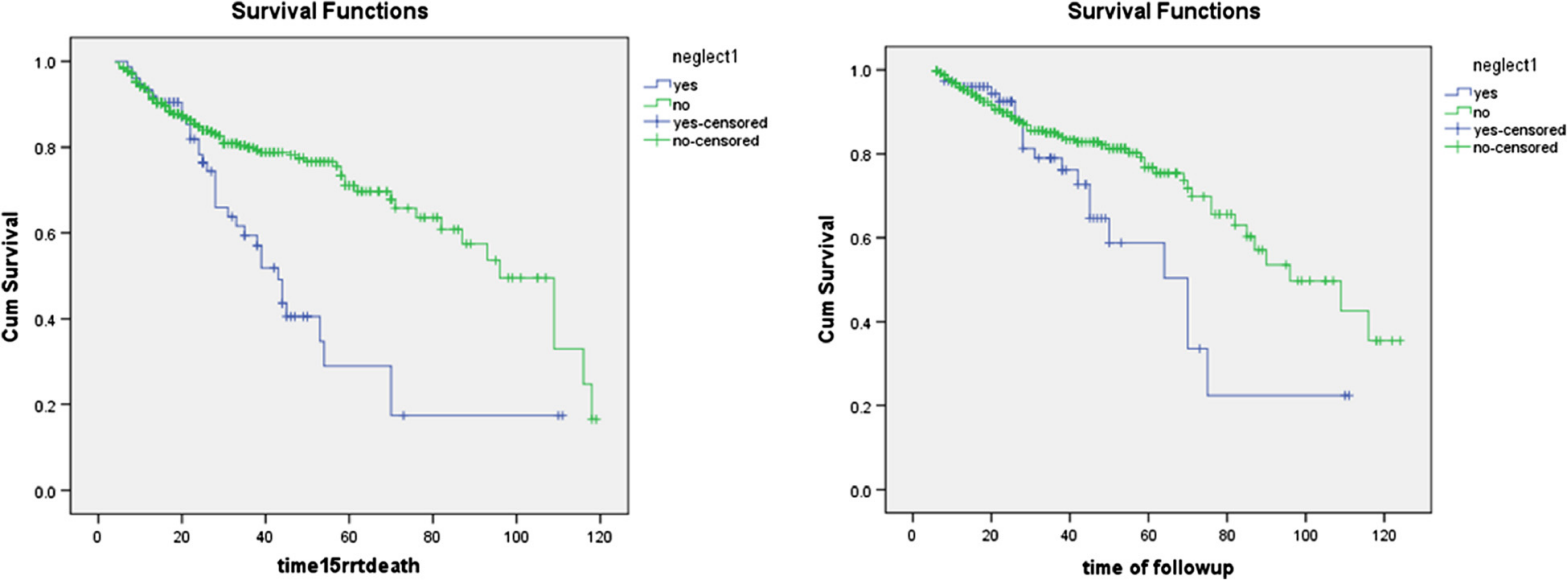
**Kaplan**-**Meier estimated survival curves.** Patients who were under medical supervision (n=389) showed a higher survival and longer time to achieve a eGFR of less than 15 cc/min compared with those who were not under medical observation (n=76) (34 vs 84months), according to the Log Rank test significance level (p<0.0001). Supervised patients also had less need for RRT and less death compared with controls (20 vs. 67months) (P = 0.029)

This cohort study included 465 newly diagnosed adult CKD patients who had been visited in the clinic for at least 6 months (Figure 1). The mean age of patients was 61.33±14.9 years (16–95 years). The ratio of males/females was 1.47 (277/188) and the total patient-years of follow-up was 1382.3. Overall, 13 patients died (1.1/100 person-years) and 71 patients started RRT (5.1/100 person-years) in the cohort (Table 1).

**Table 1:** Demographic and endpoint data of the two groups

	**Group1**	**Group2**	**P value**
	**Control group (76)**	**treated group (389)**	
CKD stage number(percent)			0.99
3a:60>GFR≥45	14(18.4)	140(36.0)	
3b:45>GFR≥30 number(percent)	35(46.1)	180(46.3)	
4:30>GFR≥15 number(percent)	27(35.5)	69(17.7)	
(GFRcc/min/1.73m^2^)			
CKD stage (RRT+death)/total(%)			0.048^*^
3a:	(3+0)/14(21.4)	34.66±11.03	
3b:	(9+2)/35(31.4)	(11+5)/180(8.9)	
4:	(5+1)/27(22.2)	(5+1)/69(8.7)	
GFRcc/min/1.73m^2^ mean±SD	35.09±10.56	(38+7)/140(32.1)	0.753
Age(years) mean±SD	56.5±17.3	62.3±14.2	0.008^*^
Diabetes mellitus	23(30.3%)	130(33.4%)	0.328
Sex(Male/Female)	43/33=1.3	234/155=1.5	0.324
Anemia(Hb<11gr/dl)	13/48	42/203	0.218
Systolic HTN(>14CmHg)	32/60	113/312	0.010*
Systolic Blood pressure (CmHg) mean±SD	14.91±2.85	13.82±2.68	0.005^*^
Diastolic Blood pressure (CmHg) mean±SD	8.34±1.47	7.74±1.30	0.001^*^
Hemoglobin(gr/dl) mean±SD	12.58±2.51	12.61±2.03	0.913
Calcium(mg/dl) mean±SD	9.12±0.70	9.22±1.00	0.681
Phosphorus(mg/dl) mean±SD	4.30±1.07	4.04±0.77	0.348
Uric acid(mg/dl) mean±SD	7.45±1.84	9.46±12.84	0.549
Cholesterol(mg/dl) mean±SD	209.56±57.66	196.19±69.14	0.251
Triglyceride(mg/dl) mean±SD	266.14±181.11	192.40±119.00	0.048

### Baseline data

The baseline data of patients are summarized in Table 1. The frequency of different CKD stages or diabetes as the cause of CKD was similar between the two groups. However, the mean age of patients with medical supervision was higher than that in the control group (62.3±14.2 vs 56.5±17.3 years, p=0.008). Mean systolic and diastolic blood pressure were higher in the control group than those in the supervised group.

### Follow-up data

Mean follow-up in the control group and supervised group was 33.29±20.50 months (7–111 months) and 36.03±25.24 months (6–124 months), respectively. The median follow-up in the control and supervised groups was 28 and 29 months, respectively. The laboratory data during the follow up period is presented for comparison between the two groups (Table 2).In both groups, a substantial number of patients survived without RRT after 1 year (96%).However, over time, the percentage of those who did not receive medical surveillance suffered more death or needed to start RRT compared with those who received medical service (26.3% vs 17.2%,p=0.048). Figure 1 and Table 3 show the outcomes of the entire cohort, grouped according to the stage of CKD. Five patients died during the first 2 years of follow up. Changes in mean blood pressure, hemoglobin and phosphate during follow-up are shown in Table 2. We observed that hypertension control and higher hemoglobin levels were achieved in those under medical supervision. Similarly, HBV vaccination rate in them was higher than the control group (76.8% vs 23.7%, p<0.0001).

**Table 2:** Comparison of continuous variables between the two groups

	**Neglected group**	**Under supervision**	**P value**
	**mean± SD(number)**	**mean± SD (number)**	
Hemoglobin (gr/dl)			
1st year	11.96±2.29(42)	12.45±1.86(329)	0.117
2nd year	11.56±2.28(36)	12.49±1.92 (269)	0.008^*^
3rd year	12.06±2.04(29)	12.64±1.86 (193)	0.121
4th year	11.65±2.29(24)	12.51±1.89 (134)	0.050^*^
5th year	10.35±2.51(6)	12.68±2.02 (95)	0.008^*^
Systolic Blood pressure(Cm Hg)			
1st year	14.01±2.14 (53)	13.71±4.98 (341)	0.444
2nd year	14.18±2.04 (40)	13.32±1.77 (281)	0.005^*^
3rd year	14.10±2.29 (31)	13.17±1.85 (198)	0.012^*^
4th year	14.49±2.17 (21)	13.15±1.64 (137)	0.001^*^
5th year	15.14±1.95 (7)	12.96±1.80 (100)	0.003^*^
Diastolic Blood pressure(Cm Hg)			
1st year	7.88±1.16 (53)	7.73± 4.57(341)	0.804
2nd year	8.07±1.26 (40)	7.36±0.88 (281)	0.001^*^
3rd year	8.15±1.30 (31)	7.43±0.96 (195)	0.006^*^
4th year	8.08±1.25 (21)	7.45± 0.86(137)	0.038^*^
5th year	8.00±1.04 (7)	7.40±0.88 (100)	0.085^*^
Phosphorus(mg/dl)			
1st year	4.13±0.92(38)	3.97±0.65(302)	0.339
2nd year	4.42±0.97(32)	3.97±0.70(241)	0.015^*^
3rd year	4.18±0.77(22)	4.02±1.61(175)	0.662
4th year	4.37±0.92(21)	3.94±0.58(125)	0.048^*^
5th year	4.57±1.19(7)	3.96±0.66(82)	0.228

**Table 3:** Survival table

**Time (Month)**	**Group1**			**Time (Months)**	**Group 2**		
		**Cumulative proportion surviving at time**	**RRT/ Death**	**Remaining number**	**Cumulative proportion surviving at time**	**RRT/ Death**	**Remaining number**
	**Estimated time ± SE**				**Estimated time ± SE**		
11	0.960±0.023	3/0	70	11	0.968±0.009	10/2	341
22	0.925±0.033	4/1	51	23	0.899±0.017	29/4	236
38	0.762±0.063	10/2	27	38	0.840±0.022	38/8	155
50	0.588±0.093	14/2	10	50	0.813±0.025	41/9	99
64	0.504±0.111	15/2	6	62	0.754±0.035	44/11	55
70	0.336±0.122	16/3	4	71	0.698±0.045	47/11	35

By Cox proportional hazard model, we found that the only significant baseline predictor for the need for RRT or death was baseline diastolic blood pressure (p=0.004), and hemoglobin, triglycerides and age were not significant parameters.

## Discussion

One of the main concerns of efficacy of CKD screening programs is the effect of follow-up on decreasing the speed of disease or preventing its inevitable, mainly cardiovascular complications. Our cohort showed a significantly higher survival and a longer time to show a eGFR of less than 15 cc/min in patients who had been under physician supervision compared with those without supervision. Supervised patients also had less need of RRT and less death compared with the control group. Our study confirmed that by using cheap diagnostic tests, characteristics that may predict RRT can be determined and controlled with simple clinical strategies [11]. It appears that if patients are observed by nephrologists or possibly internists, the rate of decline of kidney function can be slowed and the potential negative consequences of the disease for the individual and the health system can at least be postponed. In our study, we used the maximum level of non-compliance of patients as not referring to a physician for more than 1 year, which was equal to taking less treatment than those who visited their physician regularly, and evaluated its effect on outcome during the cohort. As expected, patient survival was similar at 96% in both groups in the first year. The probability of mortality increases with a decrease in GFR and the accumulated amount of toxic agents over time, which emphasizes the slow nature of the disease and asymptomatic early stages of CKD.

Interestingly, in our study the mortality rate was lower than that in many other studies, including Johnson et al., which found 11.4 deaths/100 person-years and 1.6 progressions to RRT/100 person-years. [11] The reason for this difference between studies may be the higher number of old patients in the previous study (56.3% versus 14% older than 75 years). Different methods of patient referral to nephrologists can play a role in difference in outcome. In Iran, a patient who is informed of a kidney problem by the physician can choose to seek advice directly from a nephrologist or go to a family physician or even ignore it. It appears that some patients die before reaching ESRD, the destination of CKD road. In agreement with this possibility is the age distribution of patients who initiate RRT in Iran. According to the ESRD registry, the mean age of men and women who started RRT was 52.5 years and 53.0 years in 2006, respectively, which is lower than many other countries with similar situation [12].

Levin et al. found that in a retrospective cohort of a provincial registry database of patients with an eGFR less than 30 cc/min/1.73 m^2^ (4231 patients) with access to medical services and a median follow-up of 31 months, there was large heterogeneity of outcome (death or start of RRT). Seventy-six percent of patients with rapid progression (annual eGFR change >5 cc/min/1.73 m^2^) started RRT but 27% of those with a slower decrease in eGFR started RRT. Interestingly, the authors showed higher systolic blood pressure and phosphorus levels, and lower calcium and albumin levels were associated with a rapid progression and time to RRT by multivariate analysis [13]. We conclude that managing the above-mentioned variables is important among those who are under medical supervision compared with those without any follow-up.

In a meta-analysis of 13 studies, as part of the process of CKD prognosis collaboration in the KDIGO conference on CKD controversies, a stronger association of lower eGFR with ESRD than mortality was found. The hazard ratios for ESRD in patients with a GFR of 30–44 cc/min/1.73 m^2^ (2.72; 95% CI: 1.29-3.37) and a GFR of 15–29 cc/min/1.73 m^2^ (10.21; 95% CI: 8.36-12.46) were significantly higher compared with that for a GFR of 45–74 cc/min/1.73 m^2^. Similar statistics for mortality in the latter two groups were 1.35 (1.23-1.49) and 2.25 (1.81-2.79), respectively [14]. An independent association of a lower eGFR with ESRD, all-cause mortality, and the risk of cardiovascular mortality has been confirmed [14,15]. However, while the Astor et al. mentioned GFR changes over time, the efficacy and components of treatment, and patients' compliance were not considered for analysis.

The current study found that treated patients with earlier stage of CKD(3a), reached endpoint (32.1%) more than control group(21.4%) , which may raise the issue of generalization, but we speculate that overlapping of the two outcomes of RRT and death is the main reason for this difference. The percentages of those who reached ESRD in stage 3a in both groups of control and treated group were 21.4% versus 21.7% (Table 1). The death rate was higher in the treated group compared with the rate in the control group, which was mainly caused by higher age of patients in the treated group.

### Study limitations

The wide standard deviation of the mean follow-up in the groups (33.29±20.50 [7–111] and 36.03±25.24 [6–124] months) was a limitation of the study, which may have had a negative effect on the internal validity of analysis. Another important point in considering the results is the higher portion of CKD stage 4 in the control group. Although this was not statistically significant, it may theoretically have increased the rate of those who reached ESRD. Furthermore, the mean age in the supervised group was significantly higher than that in the control group, which may have increased the occurrence of death.

## Conclusions

In conclusion, the results of this study show that supervision through nephrology clinics with some inexpensive protocols in patients, who desire to use such facilities, may benefit from a decrease in the rate of mortality, as well as postponement of the need for RRT.

## Competing interests

'The authors declare that they have no competing interests.

## Authors' contributions

MM-M carried out the nephrology visits. ZNH participated in the study design and performed the statistical analysis. SS-N participated in coordination of the study. All authors read and approved the final manuscript.

## Pre-publication history

The pre-publication history for this paper can be accessed here:

http://www.biomedcentral.com/1471-2369/13/138/prepub
